# *N*-Acryloylphenyl­alanine

**DOI:** 10.1107/S1600536808020849

**Published:** 2008-07-12

**Authors:** Cong-Ren Wu, Xiao-Feng Gao, Hai-Bo Wang, Dong Jin, Jin-Tang Wang

**Affiliations:** a Department of Applied Chemistry, College of Science, Nanjing University of Technology, Nanjing 210009, People’s Republic of China; b College of Chemistry and Chemical Engineering, Nanjing University of Technology, Nanjing 210009, People’s Republic of China

## Abstract

The title compound, C_12_H_13_NO_3_, was prepared by the nucleophilic substitution reaction of acryloyl chloride with glycylglycine. In the crystal structure, inter­molecular N—H⋯O, O–H⋯O and C—H⋯O hydrogen bonds link the mol­ecules into a three-dimensional network.

## Related literature

For bond-length data, see: Allen *et al.* (1987 ▶).
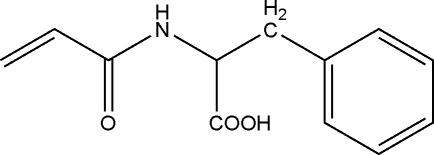


## Experimental

### 

#### Crystal data

C_12_H_13_NO_3_
*M* *_r_* = 219.23
Monoclinic, 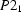

*a* = 6.0050 (12) Å
*b* = 7.5820 (15) Å
*c* = 12.512 (3) Å
β = 98.58 (3)°
*V* = 563.3 (2) Å^3^
*Z* = 2
Mo *K*α radiation
μ = 0.09 mm^−1^
*T* = 291 (2) K
0.30 × 0.10 × 0.10 mm

#### Data collection

Enraf–Nonius CAD-4 diffractometer
Absorption correction: ψ scan (North *et al.*, 1968 ▶) *T*_min_ = 0.973, *T*_max_ = 0.991
1195 measured reflections
1088 independent reflections
940 reflections with *I* > 2σ(*I*)
*R*_int_ = 0.015
3 standard reflections frequency: 120 min intensity decay: none

#### Refinement

*R*[*F* ^2^ > 2σ(*F* ^2^)] = 0.062
*wR*(*F* ^2^) = 0.161
*S* = 1.00
1088 reflections
145 parameters
1 restraint
H-atom parameters constrained
Δρ_max_ = 0.19 e Å^−3^
Δρ_min_ = −0.19 e Å^−3^

### 

Data collection: *CAD-4 Software* (Enraf–Nonius, 1989 ▶); cell refinement: *CAD-4 Software*; data reduction: *XCAD4* (Harms & Wocadlo, 1995 ▶); program(s) used to solve structure: *SHELXS97* (Sheldrick, 2008 ▶); program(s) used to refine structure: *SHELXL97* (Sheldrick, 2008 ▶); molecular graphics: *SHELXTL* (Sheldrick, 2008 ▶); software used to prepare material for publication: *SHELXTL*.

## Supplementary Material

Crystal structure: contains datablocks global, I. DOI: 10.1107/S1600536808020849/hk2488sup1.cif

Structure factors: contains datablocks I. DOI: 10.1107/S1600536808020849/hk2488Isup2.hkl

Additional supplementary materials:  crystallographic information; 3D view; checkCIF report

## Figures and Tables

**Table 1:** Hydrogen-bond geometry (Å, °)

*D*—H⋯*A*	*D*—H	H⋯*A*	*D*⋯*A*	*D*—H⋯*A*
N—H0*A*⋯O2^i^	0.86	2.30	3.036 (6)	144
O1—H1*B*⋯O3^ii^	0.82	1.84	2.614 (6)	156
C12—H12*B*⋯O1^iii^	0.93	2.60	3.178 (8)	121

Symmetry codes: (i) 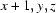; (ii) 
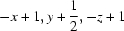
; (iii) 
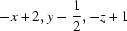
.

**Fig. 1.**
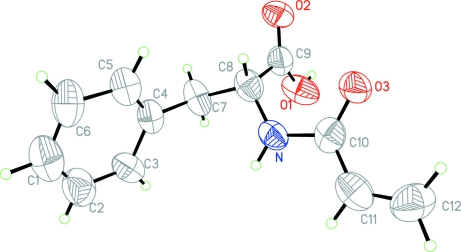
The molecular structure of the title molecule, with the atom-numbering scheme. Displacement ellipsoids are drawn at the 30% probability level.

**Fig. 2.**
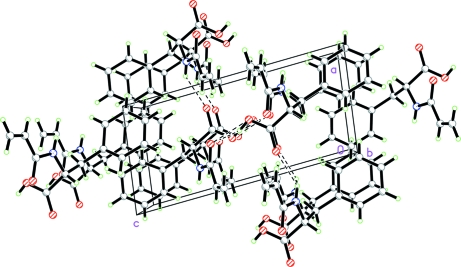
A packing diagram of the title compound. Hydrogen bonds are shown as dashed lines.

## supplementary crystallographic information

### Comment

N-Acryloylphenylalanie is one of the useful synthetic intermediates and free radical addition monomers. The crystal structure determination of the title compound has been carried out in order to elucidate the molecular conformation. We report herein its synthesis and crystal structure.

In the molecule of the title compound (Fig. 1) the bond lengths and angles are within normal ranges (Allen *et al.*,  1987).

In the crystal structure, intermolecular N-H···O, O-H···O and C-H···O hydrogen bonds (Table 1) link the molecules into a three dimensional network (Fig. 2), in which they may be effective in stabilization of the structure.

### Experimental

For the preparation of the title compound, to a well stirred solutions of phenylalanie (2.5 g) in H_2_O (30 ml) and sodium hydroxide (0.66 g) in H_2_O (5 ml), acryloyl chloride (1.34 ml) containing diphenylpicrylhydrazyl polymerization inhibitor (0.01%) and sodium hydroxide solution (0.66 g) in H_2_O (5 ml) were added dropwise simultaneously over a 30 min period and the stirring was continued for another 1 h. The reaction mixture was kept at 273 K in an ice-water bath. The solution was acidified to pH = 2 with HCl (6 N). The resulting solid was filtered off, and crystallized from ethanol (95%) (yield; 61%, m.p.401-403 K).

### Refinement

H atoms were positioned geometrically, with O-H = 0.82 Å (for OH), N-H = 0.86 Å (for NH) and C-H = 0.93, 0.98 and 0.97 Å for aromatic, methine and methylene H, respectively, and constrained to ride on their parent atoms with U_iso_(H) = xU_eq_(C,N,O), where x = 1.5 for OH H and x = 1.2 for all other H atoms.

### Crystal data

**Table:**

C_12_H_13_NO_3_	*F*_000_ = 232
*M**_r_* = 219.23	*D*_x_ = 1.293 Mg m^−^^3^
Monoclinic, *P*2_1_	Melting point: 402 K
Hall symbol: P 2yb	Mo *K*α radiation λ = 0.71073 Å
*a* = 6.0050 (12) Å	Cell parameters from 25 reflections
*b* = 7.5820 (15) Å	θ = 10–14º
*c* = 12.512 (3) Å	µ = 0.09 mm^−^^1^
β = 98.58 (3)º	*T* = 291 (2) K
*V* = 563.3 (2) Å^3^	Block, colorless
*Z* = 2	0.30 × 0.10 × 0.10 mm

### Data collection

**Table:**

Enraf–Nonius CAD-4 diffractometer	*R*_int_ = 0.015
Radiation source: fine-focus sealed tube	θ_max_ = 25.2º
Monochromator: graphite	θ_min_ = 1.7º
*T* = 291(2) K	*h* = −7→7
ω/2θ scans	*k* = 0→9
Absorption correction: ψ scan(North *et al.*, 1968)	*l* = 0→14
*T*_min_ = 0.973, *T*_max_ = 0.991	3 standard reflections
1195 measured reflections	every 120 min
1088 independent reflections	intensity decay: none
940 reflections with *I* > 2σ(*I*)	

### Refinement

**Table:**

Refinement on *F*^2^	Secondary atom site location: difference Fourier map
Least-squares matrix: full	Hydrogen site location: inferred from neighbouring sites
*R*[*F*^2^ > 2σ(*F*^2^)] = 0.062	H-atom parameters constrained
*wR*(*F*^2^) = 0.161	*w* = 1/[σ^2^(*F*_o_^2^) + (0.06*P*)^2^ + 0.62*P*] where *P* = (*F*_o_^2^ + 2*F*_c_^2^)/3
*S* = 1.01	(Δ/σ)_max_ < 0.001
1088 reflections	Δρ_max_ = 0.19 e Å^−^^3^
145 parameters	Δρ_min_ = −0.19 e Å^−^^3^
1 restraint	Extinction correction: SHELXL97 (Sheldrick, 2008)
Primary atom site location: structure-invariant direct methods	Extinction coefficient: 0.028 (5)

### Special details

**Table:**

Geometry. All e.s.d.'s (except the e.s.d. in the dihedral angle between two l.s. planes) are estimated using the full covariance matrix. The cell e.s.d.'s are taken into account individually in the estimation of e.s.d.'s in distances, angles and torsion angles; correlations between e.s.d.'s in cell parameters are only used when they are defined by crystal symmetry. An approximate (isotropic) treatment of cell e.s.d.'s is used for estimating e.s.d.'s involving l.s. planes.
Refinement. Refinement of F^2^ against ALL reflections. The weighted R-factor wR and goodness of fit S are based on F^2^, conventional R-factors R are based on F, with F set to zero for negative F^2^. The threshold expression of F^2^ > 2sigma(F^2^) is used only for calculating R-factors(gt) etc. and is not relevant to the choice of reflections for refinement. R-factors based on F^2^ are statistically about twice as large as those based on F, and R- factors based on ALL data will be even larger.

### Fractional atomic coordinates and isotropic or equivalent isotropic displacement parameters (Å^2^)

**Table:**

	*x*	*y*	*z*	*U*_iso_*/*U*_eq_	
N	0.7481 (7)	0.7059 (7)	0.2952 (3)	0.0555 (11)	
H0A	0.8730	0.7469	0.2798	0.067*	
O1	0.5602 (6)	0.8820 (8)	0.4513 (3)	0.0843 (15)	
H1B	0.4871	0.9128	0.4985	0.126*	
C1	0.8147 (14)	0.9763 (11)	−0.0922 (6)	0.086 (2)	
H1A	0.8611	0.9777	−0.1599	0.103*	
O2	0.2165 (6)	0.8618 (7)	0.3516 (3)	0.0700 (11)	
C2	0.9519 (13)	1.0469 (10)	−0.0068 (7)	0.084 (2)	
H2A	1.0883	1.0979	−0.0164	0.101*	
O3	0.5572 (6)	0.4855 (7)	0.3658 (3)	0.0632 (11)	
C3	0.8883 (9)	1.0423 (9)	0.0922 (5)	0.0699 (16)	
H3A	0.9839	1.0857	0.1517	0.084*	
C4	0.6681 (9)	0.9687 (8)	0.1060 (4)	0.0592 (13)	
C5	0.5445 (10)	0.8933 (9)	0.0224 (4)	0.0670 (15)	
H5A	0.4140	0.8336	0.0325	0.080*	
C6	0.6059 (12)	0.9014 (10)	−0.0818 (5)	0.0801 (19)	
H6A	0.5110	0.8584	−0.1417	0.096*	
C7	0.5920 (12)	0.9795 (10)	0.2154 (5)	0.0743 (17)	
H7A	0.4576	1.0520	0.2085	0.089*	
H7B	0.7079	1.0400	0.2642	0.089*	
C8	0.5411 (10)	0.8052 (8)	0.2676 (4)	0.0608 (15)	
H8A	0.4362	0.7369	0.2159	0.073*	
C9	0.4281 (9)	0.8489 (9)	0.3655 (4)	0.0634 (15)	
C10	0.7431 (9)	0.5410 (8)	0.3477 (3)	0.0566 (14)	
C11	0.9502 (11)	0.4520 (10)	0.3691 (4)	0.0701 (18)	
H11A	1.0794	0.5112	0.3564	0.084*	
C12	0.9710 (12)	0.2919 (10)	0.4055 (6)	0.083 (2)	
H12A	0.8446	0.2297	0.4189	0.099*	
H12B	1.1124	0.2392	0.4183	0.099*	

### Atomic displacement parameters (Å^2^)

**Table:**

	*U*^11^	*U*^22^	*U*^33^	*U*^12^	*U*^13^	*U*^23^
N	0.055 (2)	0.065 (3)	0.049 (2)	−0.007 (2)	0.0183 (18)	−0.002 (2)
O1	0.076 (3)	0.118 (4)	0.066 (2)	−0.021 (3)	0.033 (2)	−0.036 (3)
C1	0.118 (5)	0.070 (4)	0.076 (4)	0.005 (5)	0.036 (4)	0.008 (4)
O2	0.0515 (19)	0.084 (3)	0.078 (2)	0.001 (2)	0.0231 (17)	0.001 (3)
C2	0.093 (5)	0.074 (5)	0.090 (4)	0.000 (4)	0.029 (4)	0.023 (4)
O3	0.0551 (19)	0.090 (3)	0.0452 (18)	−0.009 (2)	0.0088 (14)	0.010 (2)
C3	0.062 (3)	0.074 (4)	0.078 (4)	−0.003 (3)	0.026 (3)	0.000 (3)
C4	0.077 (3)	0.052 (3)	0.052 (3)	0.004 (3)	0.020 (2)	0.005 (3)
C5	0.081 (4)	0.063 (4)	0.059 (3)	−0.003 (3)	0.019 (3)	0.004 (3)
C6	0.116 (5)	0.077 (5)	0.048 (3)	0.001 (4)	0.017 (3)	−0.004 (3)
C7	0.099 (4)	0.066 (4)	0.064 (3)	−0.017 (4)	0.032 (3)	0.002 (3)
C8	0.072 (3)	0.064 (4)	0.048 (3)	−0.012 (3)	0.016 (2)	−0.006 (3)
C9	0.068 (3)	0.068 (4)	0.055 (3)	−0.006 (3)	0.011 (2)	0.010 (3)
C10	0.073 (3)	0.069 (4)	0.030 (2)	0.003 (3)	0.011 (2)	−0.006 (2)
C11	0.083 (4)	0.080 (5)	0.053 (3)	−0.010 (4)	0.031 (3)	−0.014 (3)
C12	0.067 (4)	0.063 (4)	0.115 (6)	0.003 (3)	0.003 (4)	−0.014 (4)

### Geometric parameters (Å, °)

**Table:**

N—C10	1.414 (8)	C4—C7	1.509 (7)
N—C8	1.451 (7)	C5—C6	1.408 (8)
N—H0A	0.8600	C5—H5A	0.9300
O1—C9	1.261 (6)	C6—H6A	0.9300
O1—H1B	0.8200	C7—C8	1.525 (9)
C1—C2	1.358 (11)	C7—H7A	0.9700
C1—C6	1.400 (10)	C7—H7B	0.9700
C1—H1A	0.9300	C8—C9	1.523 (7)
O2—C9	1.261 (6)	C8—H8A	0.9800
C2—C3	1.350 (10)	C10—C11	1.405 (9)
C2—H2A	0.9300	C11—C12	1.296 (10)
O3—C10	1.245 (6)	C11—H11A	0.9300
C3—C4	1.468 (8)	C12—H12A	0.9300
C3—H3A	0.9300	C12—H12B	0.9300
C4—C5	1.319 (8)		
			
C10—N—C8	119.5 (4)	C4—C7—H7A	108.1
C10—N—H0A	120.2	C8—C7—H7A	108.1
C8—N—H0A	120.2	C4—C7—H7B	108.1
C9—O1—H1B	109.5	C8—C7—H7B	108.1
C2—C1—C6	122.2 (6)	H7A—C7—H7B	107.3
C2—C1—H1A	118.9	N—C8—C9	112.9 (5)
C6—C1—H1A	118.9	N—C8—C7	109.4 (5)
C3—C2—C1	119.4 (7)	C9—C8—C7	107.3 (5)
C3—C2—H2A	120.3	N—C8—H8A	109.0
C1—C2—H2A	120.3	C9—C8—H8A	109.0
C2—C3—C4	120.0 (6)	C7—C8—H8A	109.0
C2—C3—H3A	120.0	O2—C9—O1	126.5 (5)
C4—C3—H3A	120.0	O2—C9—C8	117.8 (5)
C5—C4—C3	118.8 (5)	O1—C9—C8	115.4 (5)
C5—C4—C7	122.2 (5)	O3—C10—C11	126.5 (6)
C3—C4—C7	119.0 (5)	O3—C10—N	117.7 (5)
C4—C5—C6	121.4 (6)	C11—C10—N	115.7 (5)
C4—C5—H5A	119.3	C12—C11—C10	123.6 (7)
C6—C5—H5A	119.3	C12—C11—H11A	118.2
C1—C6—C5	117.7 (6)	C10—C11—H11A	118.2
C1—C6—H6A	121.1	C11—C12—H12A	120.0
C5—C6—H6A	121.1	C11—C12—H12B	120.0
C4—C7—C8	116.7 (6)	H12A—C12—H12B	120.0
			
C6—C1—C2—C3	1.3 (12)	C10—N—C8—C7	178.2 (4)
C1—C2—C3—C4	−2.8 (11)	C4—C7—C8—N	67.3 (7)
C2—C3—C4—C5	6.2 (10)	C4—C7—C8—C9	−169.9 (5)
C2—C3—C4—C7	−174.7 (7)	N—C8—C9—O2	−149.5 (6)
C3—C4—C5—C6	−8.0 (10)	C7—C8—C9—O2	89.9 (7)
C7—C4—C5—C6	172.9 (7)	N—C8—C9—O1	35.8 (8)
C2—C1—C6—C5	−2.9 (12)	C7—C8—C9—O1	−84.9 (7)
C4—C5—C6—C1	6.5 (10)	C8—N—C10—O3	1.8 (6)
C5—C4—C7—C8	58.1 (9)	C8—N—C10—C11	178.5 (4)
C3—C4—C7—C8	−121.1 (7)	O3—C10—C11—C12	4.3 (9)
C10—N—C8—C9	58.7 (6)	N—C10—C11—C12	−172.1 (6)

### Hydrogen-bond geometry (Å, °)

**Table:**

*D*—H···*A*	*D*—H	H···*A*	*D*···*A*	*D*—H···*A*
N—H0A···O2^i^	0.86	2.30	3.036 (6)	144
O1—H1B···O3^ii^	0.82	1.84	2.614 (6)	156
C12—H12B···O1^iii^	0.93	2.60	3.178 (8)	121

Symmetry codes: (i) *x*+1, *y*, *z*; (ii) −*x*+1, *y*+1/2, −*z*+1; (iii) −*x*+2, *y*−1/2, −*z*+1.
